# Evaluation of antimicrobial activity against *Enterococcus faecalis* of activated chelating agents in different final rinse protocols: An *ex vivo* study

**DOI:** 10.4317/jced.59547

**Published:** 2022-08-01

**Authors:** Adriana Oliveira, Daniel Rocha, Alexandre De Martin, Ana-Grasiela Limoeiro, Wayne Nascimento, Carlos Fontana, Rina Pelegrine, Elizabeth Martinez, Carlos Bueno

**Affiliations:** 1 Department of Endodontics, Faculdade de Ilhéus Bahia, Brazil

## Abstract

**Background:**

To evaluate the antimicrobial activity of the following chelating agents against Enterococcus faecalis using quantitative polymerase chain reaction (qPCR) analysis: 1% peracetic acid (PA), 1% peracetic acid with 0.1% cetrimide (PAC), and 17% ethylenediaminetetraacetic acid (EDTA) activated by passive ultrasonic irrigation (PUI) or with Easy Clean (EC), all followed by 2.5% sodium hypochlorite (NaOCl).

**Material and Methods:**

A total of 80 permanent human mandibular premolars were randomly divided into eight experimental groups according to the chemical solution and agitation protocol used: Group PA + PUI; Group PA + EC; Group PAC + PUI; PAC + EC; group EDTA + PUI; EDTA + EC, all followed by 2.5% NaOCl; and two control groups with saline solution(NaCl): NaCl + PUI and NaCl + EC. Microbial samples were collected before (S1) and after the irrigation protocol (S2). Intracanal E. faecalis reduction analysis was performed by qPCR. Intragroup analysis was performed using the Wilcoxon signed-rank test for paired data, and intergroup analysis was performed using the Wilcoxon Mann-Whitney U test for independent samples. The significance level was set at *p*< 0.05.

**Results:**

A significant difference was found between S1 and S2 in all groups except NaCl+ EC (*p* = 0.1602). Comparison between groups showed that PAC + PUI was significantly different from PA +EC (*p* = 0.0448).

**Conclusions:**

The activated chelating agents were effective against *E. faecalis*, with significant results compared to the control groups. The peracetic acid with cetrimide activated by PUI showed better results than peracetic acid with EC.

** Key words:**Easy clean, Final irrigation protocols, Passive ultrasonic irrigation, Peracetic acid.

## Introduction

A disadvantage of the speed of root canal instrumentation sequences is their negative influence on the time-dependent properties of the irrigants (1). To address this issue, final irrigation protocols (FIPs) have been developed to optimize antimicrobial activity, smear layer removal (SL), wettability, and ability to reach the most complex areas of canal anatomy (2-5). In general, these protocols propose alternating irrigation with chelating and biocidal agents activated manually or by sonic, ultrasonic, or mechanical methods (5).

One of the most used procedures in the clinic is a combination of NaOCl and 17% EDTA, which combines the antimicrobial and organic matter dissolving properties of NaOCl with the chelating effect of EDTA (1). However, this mixture is associated with erosion and a reduction in dentin microhardness, and it lacks residual antimicrobial activity (3,6).

Peracetic acid (PA) has been investigated as an alternative single rinse agent for use during chemical mechanical preparation (CMP) (7). It has antimicrobial properties (2,8) and the ability to remove SL and has shown favorable results even at lower concentrations (7,9).

Review of the literature did not find any published studies investigating whether the addition of a surfactant to PA has a positive effect on these properties, including evaluation of the influence of different methods of activating the rinse. Cetrimide (C) or cetrimonium is a cationic surfactant that not only reduces surface tension, but also has bactericidal effects (3,10,11), can reduce biofilm mechanical stability (12), and has residual antimicrobial activity (3).

Among the available methods for irrigation activation, PUI has the potential to remove debris, organic tissue, and biofilm in areas inaccessible to instrumentation due to cavitation and microacoustic flow of the irrigation fluid (2,4-6)

Easy Clean (EC) is a plastic instrument made of acrylonitrile butadiene styrene with a diameter and taper of 25.04 and an “airplane wing” shaped cross section. It was originally recommended for reciprocal use but has been found to maintain the same efficiency with continuous rotation. EC can be used up to the working length (WL) to facilitate the contact of the irrigation solution in the apical part of the root canal system (4,13,14).

In this context, the present study investigated the activity of the chelating agents PA (1%), PAC (1%/0.1%), and EDTA (17%), all followed by NaOCl (2.5%), using two different methods of agitation (PUI or EC) by molecular microbiological qPCR analysis against *E. faecalis*. The null hypothesis was that there would be no difference in antimicrobial activity against *E. faecalis* between the solution types and agitation methods tested.

## Material and Methods

After approval of the study protocol by the institutional research ethics committee (number: 3.404.272), 80 permanent human mandibular premolars were select, all of which had been recently extracted for therapeutic reasons.

The teeth with single roots and a curvature angle of < 10 using the Schneider method (15), were cleaned with a T1-S tip in an ultrasonic device (Schuster, Santa Maria, RS, Brazil) to remove all remaining debris and ligament tissue, and then stored in 0.1% thymol solution (Ao Pharmacêutico, Maceio, AL, Brazil) until use.

The sample size was calculated based on a previous study (2) and resulted in 10 samples per experimental group (n=10). For this purpose, an analysis of variance (ANOVA) was used with a minimum difference between treatment means of 0.10, a standard deviation of error of 0.056, a statistical power of 0.80 and an alpha of 0.05.

After removal of thymol debris with saline, teeth were radiographed in the buccolingual and mesiodistal directions. The following inclusion criteria were applied: Absence of fractures and calcifications; a single, straight root canal with an oval cross-section (buccolingual length = twice the mesiodistal length, 3 mm from the canal opening); and no previous endodontic treatment.

The crowns of all teeth were cut near the enamel-cement junction with a double-sided diamond disk (Microdont, São Paulo, SP, Brazil) to obtain a 16-mm root segment measured with a digital caliper (Nove54, Curitiba, PR, Brazil). The WL was determined by advancing a #10 K-file (Angelus, Londrina, PR, Brazil) with an operating microscope (DFVasconcellos, Valen , RJ, Brazil) under 12.5x magnification until it was visible at the apical foramen and then subtracting 1 mm from the resulting length.

To prevent leakage of the irrigation fluid, the roots were impregnated with two layers of epoxy resin (Araldite, Brascola Ltda., Tabo  da Serra, SP, Brazil), with the second layer applied after a 30-minute curing time, and with an additional layer of utility wax (Lysanda Ltda, São Paulo, SP, Brazil) on the apical part. They were then placed in Eppendorf polypropylene tubes (Eppendorf do Brasil Ltda., São Paulo, SP, Brazil) filled with condensation silicone (Perfil, Coltene Brasil, Rio de Janeiro, RJ, Brazil) and surrounded by a thin layer of cyanoacrylate adhesive (Super Bonder, Henkel, Jundiaí, SP, Brazil) to ensure stability (4).

The chemical mechanical preparation was performed by a single operator using reciprocal kinematics. Briefly, a glide path was created using #10 and #15 K files along the entire length. Then, a ProDesign R 35.05 file (Easy Equipamentos Odontológicos, Belo Horizonte, MG, Brazil), driven by the Easy SI electric motor (Easy Equipamentos Odontológicos, Belo Horizonte, MG, Brazil), was used in ProDesign R mode to quarter the canal until the WL preparation was completed. At this stage, patency was maintained with a #10 K-file advanced 1 mm beyond the apical foramen to prevent obstruction by debris. Irrigation was performed by the conventional positive pressure method using a 5-mL disposable syringe (BD, Curitiba, PR, Brazil) and a NaviTip 30G needle (Ultradent Products Inc., South Jordan, UT, USA) advanced to 2 mm in front of WL with 2 mL of 2.5% NaOCl solution (Ao Pharmacêutico, Maceio, AL, Brazil) at the beginning of instrumentation and after each use of the files.

The smear layer was removed with 2 mL of 17% EDTA (Ao Pharmacêutico, Maceio, AL, Brazil), shaken with a FM cone (Maillefer-Dentsply, Ballaigues, Switzerland) calibrated to #35 for 3 minutes, followed by 2 mL of 2.5% NaOCl and drying with #35 absorbent paper tips (Maillefer-Dentsply, Ballaigues, Switzerland).

Instrumented roots were autoclaved at 121◦C for 15 minutes. The efficacy of sterilization was verified using samples collected with absorbent paper tips, and the turbidity of the culture medium was assessed after 24 hours. Roots were then inoculated with *E. faecalis* (ATCC-29212), cultured, and stored in liquid BHI medium containing 20% glycerol. Twenty-four hours before the experiment, 10 μL of the microorganism culture was inoculated into 5 mL of BHI culture medium and incubated at 370C for 24 hours (4).

After the incubation period, a suspension was prepared with a turbidity standard of 10.0 McFarland. Using the polypropylene tubes arranged in a cell culture plate, 20 μL of the suspension was carefully transferred into the root canals of the prepared teeth using syringes and insulin needles. Sterile cotton balls moistened with sterile distilled water were placed in four wells of the cell culture plate to ensure ambient humidity, and the plate was incubated at 370C for 21 days. BHI broth (20 μL) was added to the root canals every 24 hours to maintain microbial viability (4).

Samples were numbered and randomly divided into eight experimental groups consisting of different combinations of chelating agents and agitation methods (PUI or EC), namely: 1% peracetic acid (Dinâmica Química Contemporânea, Diadema, MG, Brazil) - PA + PUI; PA + EC; 1% peracetic acid plus 0.1% cetrimide (Dinâmica Química Contemporânea, Diadema, MG, Brazil) - PAC + PUI; PAC + EC; 17% EDTA – EDTA + PUI; EDTA + EC; and two control groups with NaCl 0.9% (Equiplex, Aparecida de Goiânia, GO, Brazil) as rinsing agent – NaCl + PUI and NaCl + EC.

After the contamination phase, the cell culture plate was opened in a laminar flow chamber (Veco, *Pi*racicaba- SP, Brazil) and a first contamination sample (S1) was obtained before applying the different FIPs. A sterile #35 paper tip was inserted into the canal and held for 1 minute (4,8). Then, with the help of sterile cotton forceps, it was transferred into a polypropylene Eppendorf tube containing 1 mL of sterile 0.9% NaCl, vortexed for 30 seconds (AP 56, Phoenix, Araraquara, SP, Brazil) and numbered as a sample.

An individual kit of instruments and materials was prepared for each FIP tooth. A 6-mL aliquot (14) of the test solution was drawn up into a 10-mL disposable syringe with NaviTip 30G needle (2 mm from WL).

Irrigation was performed by distributing 2 mL of the test solution for each 20-second shaking cycle, for a total of 3 shaking cycles per insert/tooth (4,14). Aspiration was performed using capillary tips (Ultradent products, South Jordan, UT, USA) connected to a maximum-power portable vacuum pump (Aspiramax Ma520 NS, São Paulo, SP, Brazil).

The ultrasound device chosen for PUI was the E1-Irrisonic (Helse Ultrasonic, Ribeirão Preto- SP, Brazil) coupled to a Wak U 600 ultrasound unit (Wak, Campo Belo- MG, Brazil) operating at 10% power (6,14).

EC (Easy Equipamentos Odontológicos, Belo Horizonte, MG, Brazil) was used with an X-Smart motor (Dentsply-Sirona, Ballaigues, Switzerland) in WaveOne mode in reciprocating motion (14). Both devices were inserted 2 mm short of the WL (13). At the end of each protocol, a final irrigation with 2mL of 2.5% NaOCl followed by 5 mL of 0.9% NaCl was performed. In the control groups, after three cycles of shaking with 2 mL of 0.9% NaCl, the final rinse was performed with 5 mL of saline alone. A new contamination sample was then collected (S2), using the same procedure as S1.

After collection of the biological material, the samples were stored at -200C until qPCR was performed to quantify the E. faecalis load in S1 and S2. Analyzes were performed using the Maximum SYBR Green/ ROX qPCR Master Mix detection system (Sinapse Biotecnologia Ltda, São Paulo, SP, Brazil) in a 7500 Fast Real Time PCR System (Applied Biosystems, Foster City, USA).

-Statistical analysis

The qPCR data were log-transformed and analyzed in R software (Lucent Technologies, Louisville, KY, USA). The significance level was set at 5%. The Wilcoxon signed-rank test for paired data was used for comparison between contamination samples (since there were two measurements for the same sampling unit, S1 and S2), while the Wilcoxon Mann-Whitney U test was used for comparison between groups.

## Results

All tests test the null hypothesis (H0) that there is no statistically significant difference between the compared groups. In this study, the Wilcoxon Mann-Withney U test was used for the comparison between 02 groups because it is a nonparametric test in which no normality assumption is made for the data (the test is based on the sum of the ranks of the variables). Nonparametric tests are recommended for studies with a small sample size, usually ≤30, and whose normality assumption is not met.

Descriptive analysis of the quantification of *E. faecalis* in the initial and final samples showed a significant difference between S1 and S2 in the groups PA + PUI (*p* = 0.0020), PA + EC (*p* = 0.0020), PAC + PUI (*p* = 0.0020), PAC + EC (*p* = 0.0020), EDTA + PUI (*p* = 0.0020), EDTA + EC (*p* = 0.0020), NaCl + PUI (*p* = 0.0273), except NaCl + EC (*p* = 0.1602) (Table 1).

**Table 1:**
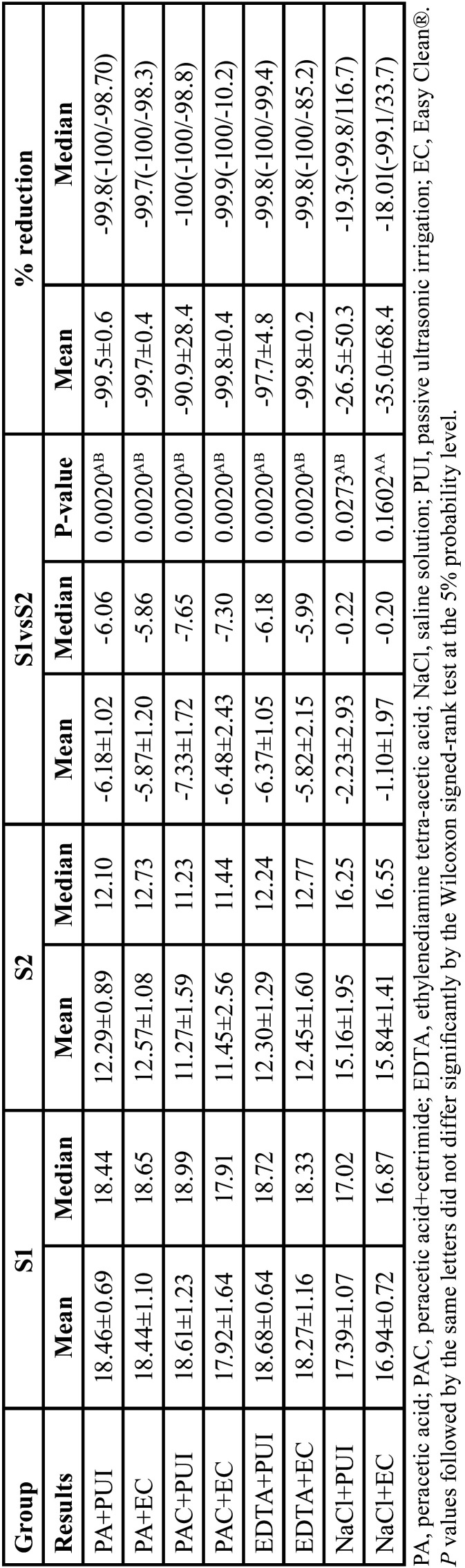
Descriptive data for phases S1 and S2 (log-transformed), difference and comparison between phases (Wilcoxon signed-rank test for paired data) and percent reduction (mean, median, minimum, and maximum).

The comparison between the different final irrigation protocols showed a significant difference between the two control groups (*p* < 0.05), which were not significantly different from each other (*p* > 0.05). There was a significant difference between PAC + PUI and PA + EC (*p* = 0.0448) (Table 2).

**Table 2:**
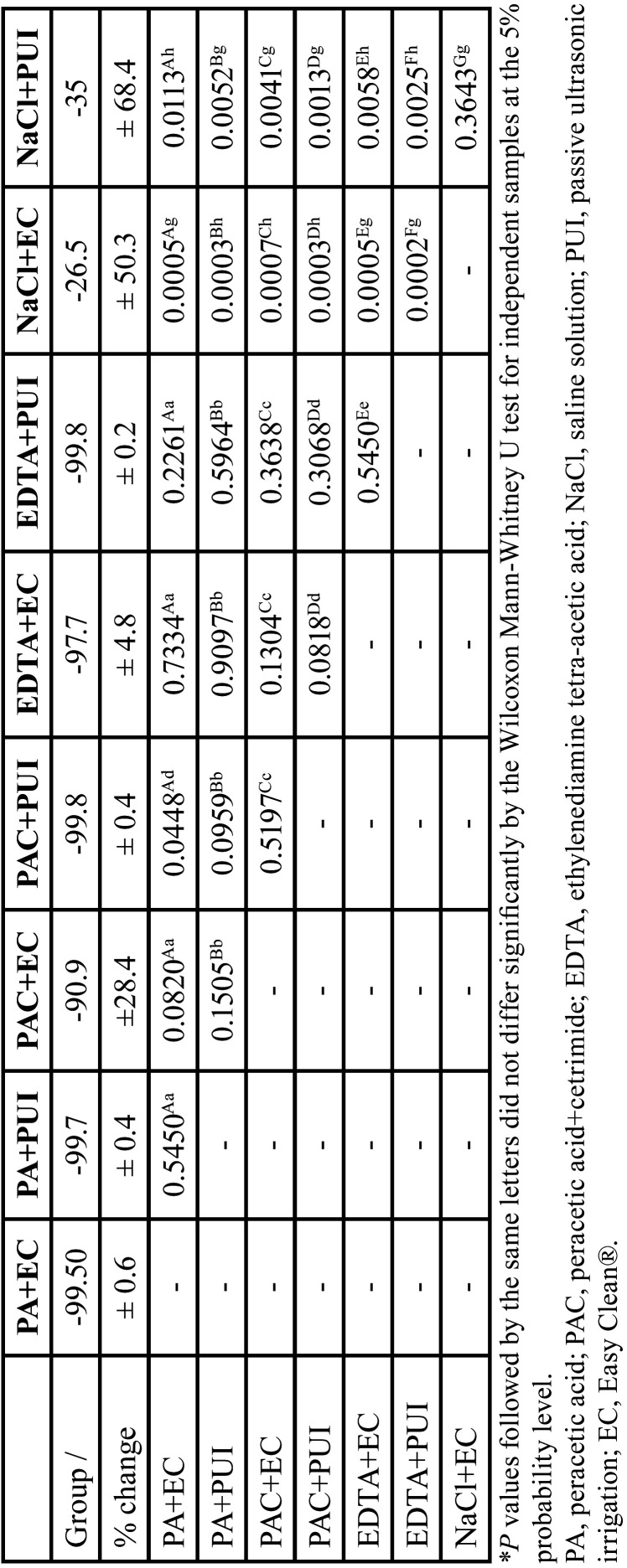
Comparisons between groups (Wilcoxon Mann-Whitney U test for independent samples).

## Discussion

Ideally, the acid or chelating agent selected for irrigation should have biocidal activity (2,6) and residual effect (3,6,16) after removal of the SL and exposure of the contaminated dentinal tubules without unduly affecting the dentin microstructure (7,14). Previous studies have shown that EDTA alone (without NaOCl), agitated manually or by PUI, had limited (5,9) or with insignificant residual effect in reducing *E. faecalis* in biofilm study models (3,16). EDTA has high surface tension and low permeability, which limits its antimicrobial depth effect (16). However, it has been shown to promote biofilm dispersion (17) and alter bacterial cell membrane permeability (12).

PA is a disinfectant widely used in the food industry and hospitals (18). Previous studies have demonstrated its ability to remove SL (7,9) and its antimicrobial activity (2,8,18), even in the presence of organic material (18). The concentration of 1% was chosen for this experiment because more concentrated solutions can lead to dentin erosion, corrosive effects on the mucosa, and greater cytotoxicity (2,8,9,18,19). In aqueous solution, PA is in equilibrium with hydrogen peroxide, acetic acid, and acetyl hydroperoxide (9). It is probably the acetic acid content that is responsible for its effect on SL. It forms complexes with calcium, which are readily soluble in water (9). Its oxidizing effect leads to denaturation of proteins, rupture of cell membrane and oxidation of sulfhydryl and formation of sulfur bonds in proteins, enzymes, and other metabolites (8). In tests with human fibroblasts, 1% PA proved to be less cytotoxic than 2.5% NaOCl and 17% EDTA (19).

In the PAC group, the detergent was added to decrease surface tension (3,10,11) and increase bactericidal activity, as shown in previous studies in which cetrimide was conjugated with other acids (3,10-12,16).

Another antimicrobial advantage of this proposed combination is the ability of cetrimide to dissolve biofilm (12) and its residual antimicrobial activity when used alone or in combination (3,16). The addition of cetrimide to EDTA and citric acid did not affect their demineralizing effects (21).

The complementary effect of NaOCl (disinfection of the dentinal tubules and removal of organic components from the SL) is essential due to the limited effect of chelators on biofilm and organic material (1,2).

However, it may directly affect the collagen previously exposed by the demineralizing agent, which affects its interaction with the adhesive agents (19). In this study, NaOCl was used at the end of the chelator activation protocols, in a low volume and without activation cycles. Nevertheless, a percentage reduction of almost 100% was achieved in S2 compared to S1, including the EDTA groups.

The cavitation and microacoustic flow effects of PUI (2,5) are highly dependent on the power of the device, the free space in the canal, and the absence of interference from the instrument tip. In this study, the E1 insert was advanced up to 2 mm short of WL (14). This is based on a previous study that showed better cleaning of the apical third when the tip of the insert was 1 and 2 mm short of WL (13).

EC is a flexible plastic instrument that favors contact with root canal walls, destroys biofilm, and displaces tissue debris (4,14,15). In this study, EC was used with reciprocal kinematics and placed 2 mm to WL, just like the E1 insert. This gave similar results to PUI as shown in previous studies (4,13,14,22).

When comparing the results between samples S1 and S2, a statistically significant reduction in microbial load was observed, except for the group in which NaCl was stirred up by the EC device. In this comparison, NaCl, although it had no antimicrobial effect, also achieved a significant reduction in bacterial load when agitated with PUI (*p* = 0.0273). This finding is consistent with previous results in which agitation with ultrasound was able to reduce the microbial load even when inert rinsing agents were used (23).

As for the comparison between the rinsing methods used and the percentage changes, a median reduction in microbial load of almost 100% was observed in the groups using activated chelating agents followed by NaOCl. These reductions were significant (*p* < 0.05) in the comparisons with the control groups (NaCl + PUI and NaCl + EC), which is consistent with previous studies using 17% EDTA in combination with NaOCl (4,6,8) and with PA at 1% (8) and 0.5% (2). There was also a significant difference between the PA + EC and PAC + PUI groups, with a greater percentage reduction in the latter (*p* = 0.0448). Therefore, the null hypothesis was rejected.

The antimicrobial role of the tested solutions was significant for the obtained results, which is consistent with previous findings (2). A similar behavior was also observed for the two stirring methods when comparing the percentage reduction within the control group.

The present study has some limitations. Collection of bacteria from the root canals using absorbent papers cones does not allow collection of bacteria within the dentinal tubules (2). Another limitation is the collection of bacteria after the irrigation protocol has been performed only once. Studies show that positive cultures can be presented up to 7 days after irrigation due to recolonization of the canal by bacteria remaining in the dentinal tubules (19). In addition, *E. faecalis* can reach a viable but non-culturable state under unfavorable conditions (24).

Further studies should evaluate the possibility of a residual effect of the PAC combination as well as its effectiveness in removing the SL, its effect on dentin microstructure and its cytotoxicity, as good results in these aspects would be extremely valuable for a final rinsing protocol.

## Conclusions

All FIPs tested in this study achieved a reduction in microbial load, except for the group in which NaCl was agitated by the device EC. The combinations PA, PAC and EDTA + NaOCl were all effective against *E. faecalis*, with both mechanical agitation methods achieving significant results compared to the control groups. Among the experimental groups, the PAC + PUI protocol also achieved significant results compared to the PA + EC protocol.
